# Feasibility study on the verification of actual beam delivery in a treatment room using EPID transit dosimetry

**DOI:** 10.1186/s13014-014-0273-8

**Published:** 2014-12-04

**Authors:** Tae Seong Baek, Eun Ji Chung, Jaeman Son, Myonggeun Yoon

**Affiliations:** Department of Bio-convergence Engineering, Korea University, Jeongneungro 161, Seongbuk-gu, Seoul 136-703 Korea; Department of Radiation Oncology, National Health Insurance Co. Ilsan Hospital, Ilsan, Korea

**Keywords:** Transit dose, EPID, Intensity modulated radiation therapy, Gamma index

## Abstract

**Purpose:**

The aim of this study is to evaluate the ability of transit dosimetry using commercial treatment planning system (TPS) and an electronic portal imaging device (EPID) with simple calibration method to verify the beam delivery based on detection of large errors in treatment room.

**Methods and materials:**

Twenty four fields of intensity modulated radiotherapy (IMRT) plans were selected from four lung cancer patients and used in the irradiation of an anthropomorphic phantom. The proposed method was evaluated by comparing the calculated dose map from TPS and EPID measurement on the same plane using a gamma index method with a 3% dose and 3 mm distance-to-dose agreement tolerance limit.

**Results:**

In a simulation using a homogeneous plastic water phantom, performed to verify the effectiveness of the proposed method, the average passing rate of the transit dose based on gamma index was high enough, averaging 94.2% when there was no error during beam delivery. The passing rate of the transit dose for 24 IMRT fields was lower with the anthropomorphic phantom, averaging 86.8% ± 3.8%, a reduction partially due to the inaccuracy of TPS calculations for inhomogeneity. Compared with the TPS, the absolute value of the transit dose at the beam center differed by −0.38% ± 2.1%. The simulation study indicated that the passing rate of the gamma index was significantly reduced, to less than 40%, when a wrong field was erroneously irradiated to patient in the treatment room.

**Conclusions:**

This feasibility study suggested that transit dosimetry based on the calculation with commercial TPS and EPID measurement with simple calibration can provide information about large errors for treatment beam delivery.

## Introduction

The goal of radiotherapy is to deliver a therapeutic dose to a tumor volume while minimizing doses to surrounding organs [1-3]. Efforts to achieve this goal have led to increasingly complex radiation delivery and dose calculation algorithms. In addition, more advanced treatment techniques, including intensity-modulated radiotherapy (IMRT), helical tomotherapy (TOMO), volumetric modulated arc therapy (VMAT) and heavy ion therapy, have been developed to overcome the disadvantages of conventional three-dimensional conformal radiotherapy (3D-CRT). Although highly technological treatment methods are important to achieve the goal of radiotherapy, these methods can be useless or even harmful to patients if the therapeutic dose is not accurately delivered as planned. For example, an IMRT planning error in New York in 2005 caused a fatal radiation overdose resulting in the death of the patient [4]. This accident suggests that accurate verification of the dose delivered to the patient is essential for maximum treatment efficacy and to prevent accidental overdoses.

Accidental exposure during radiotherapy may be avoided by learning from previous accidents [4-7]. Most of these accidents were due to human errors, including mistakes, procedural violations and inadequate procedures, all of which should be expected. These kinds of errors may be prevented by the application of several types of preventive actions during radiation treatment. One of the most important is patient-specific quality assurance (QA), which is generally performed to assure that there is no difference between the dose calculated by the TPS and the actual measured dose. Conventional patient-specific QA for IMRT involves measuring the absolute dose as well as measuring the two-dimensional dose distribution using a homogeneous phantom. EPID based portal dosimetry has recently become popular for patient-specific IMRT dose verification in radiotherapy, although EPID is the primary tool used to verify patient positioning in the treatment room [8,9].

Good agreement based on conventional QA, however, does not guarantee the accuracy of the actual dose distribution to the patient in the treatment room. In general, unexpected errors during beam delivery are difficult to detect by conventional QA, since the latter is basically pretreatment verification. A possible approach to detect errors during treatment is a transit dosimetry acquired using EPID in the treatment room. So far, various studies have been carried out for the transit dosimetry at the position of the EPID behind a patient [10-15]. Among them the use of the TPS can be considered as one of the simple ways to conduct the 2D transit dosimetry since the transit dose is easily calculated by commercial software. McNutt et al. used the convolution/superposition method to predict the dose at the level of imaging device and found that the calculated doses at the EPID level were within 4% of the measured doses in the central region of the field [13]. More recently, Reich et al. investigated the calculation of transit dose maps using commercial TPS (Pinnacle Version 6.2b, Phillips Medical System, Milpitas, USA) and reported that the calculated transit dose maps agrees well with the measured dose maps showing less than 2% of dose difference on the central beam axis [11].

Although the previous studies showed that 2D transit dosimetry based on TPS calculation and EPID measurement is simple and advantageous, more studies are needed for this method due to several reasons. First, the accurate calibration of EPID for various factors is still too complex to be adapted easily in clinic. Second, as time goes on, various TPSs are available and the algorithm of TPS becomes more accurate. Therefore, it is meaningful to test the recent TPS for the use of transit dosimetry. Third, only limited clinical cases were examined in previous studies suggesting the need of more research for the use of TPS in 2D transit dosimetry. In this study, we used EPID to measure the transit dose passing through an anthropomorphic phantom in the treatment room based on simple calibration method and evaluated the ability of transit dosimetry using commercial TPS to verify the beam delivery during treatment in radiotherapy.

## Methods and materials

**Figure 1:**
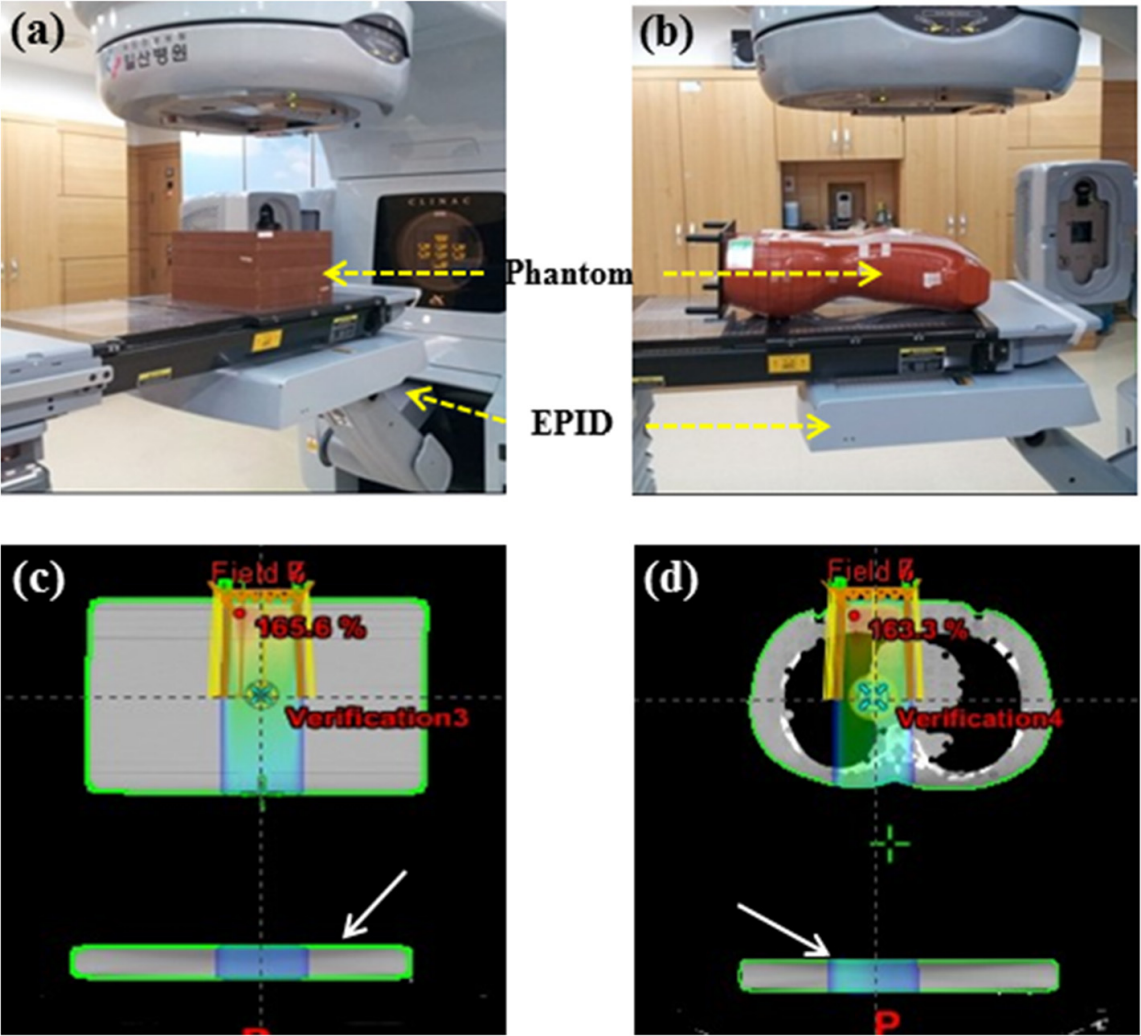
**Pictures of the experimental setup and axial views of IMRT calculations maps. (a)** a homogeneous plastic phantom, **(b)** an anthropomorphic phantom, **(c)** a homogeneous plastic phantom and **(d)** an anthropomorphic phantom.

We evaluated 24 IMRT fields used in the radiotherapy of 4 randomly selected lung cancer patients (see Table 1). All IMRT QA was carried out with the sliding window technique, after modifying the gantry angles of all treatment fields to 0 degrees. The patient-specific planned dose distributions were calculated using the Eclipse treatment planning system Version 8.0.3 (Varian Medical Systems, Salt Lake City, UT, USA) with AAA algorithm. Figure 1(a) and (b) show pictures of the experimental setup with a *homogeneous* solid water phantom (Plastic Water, New York, USA) and an *inhomogeneous* anthropomorphic phantom (Rando, NY, USA), respectively. Using the CT (LightSpeed RT16 CT-Simulator, GE Healthcare, Milwaukee, WI, USA) scanned phantom, an IMRT QA plan used for transit dosimetry was made in the TPS for selected fields. Figure 1(c) and (d) show examples of the axial view of the transit dose passing through a homogeneous plastic water phantom and anthropomorphic phantom, respectively, as calculated by the TPS.

**Table 1:** **Patient characteristics, prescribed radiation dose and fraction size**

**Patient no**	**PTV (ml)**	**Number of fields**	**Number of fractions**	**Prescribed dose (cGy)**
1	19	6	32	6400
2	31	6	27	5400
3	57	6	33	6600
4	23	6	34	6800

The transit dose maps passing through the phantoms were calculated by Eclipse TPS. To do that, we first built the artificial phantom (see arrows in Figure 1(c), (d)) at the location of EPID from TPS and ran the calculation to get the dose map in the EPID plane. In the treatment room, the radiation fields of the IMRT QA plans were delivered using a Varian Clinac iX linear accelerator (Varian Medical Systems, Palo Alto, CA) equipped with an aS1000 EPID. The EPID system is an amorphous silicon flat panel imager, with an active imaging area of 30 × 40 cm^2^ with a matrix size of 1024 × 768 pixels.

**Figure 2:**
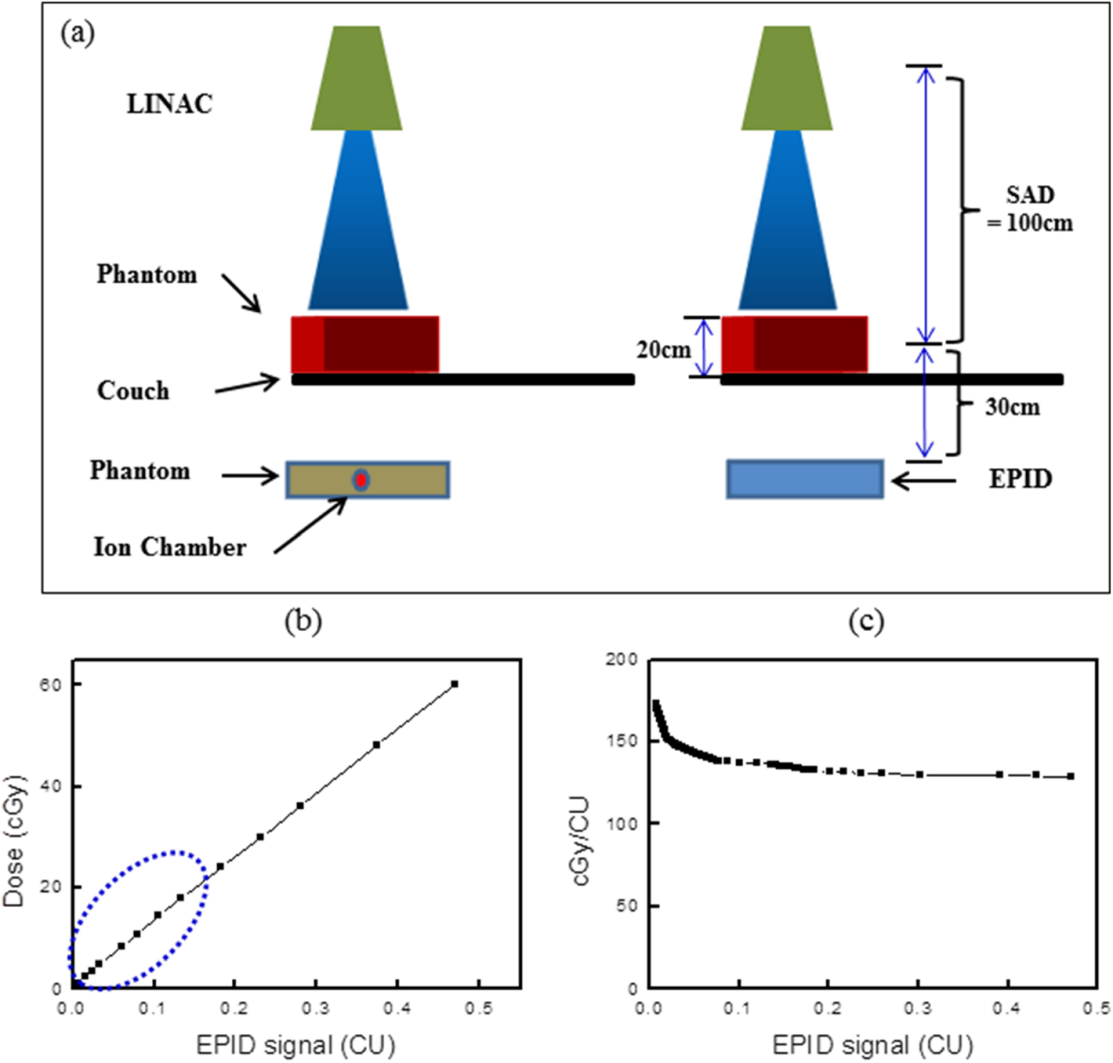
**Experimental setup for EPID and dose calibration. (a)** EPID calibration with ion chamber measurement, **(b)** radiation dose as a function of EPID in calibrated units and **(c)** dose conversion factor for EPID signals and CU.

For image acquisition and transit dose analysis, the EPID was first calibrated according to the vendor’s guidelines with dark field, flood field and the diagonal profile correction which was measured at d_max_ in water for a 40 × 40 cm^2^ open field. Then, EPID response was scaled such that 1 Calibrated Unit (CU) corresponds to 100 MU delivered by a 10 × 10 cm^2^ open field at 100 cm source-to-detector distance (SDD). It has been reported that the mechanical parts of the EPID produces a non-uniform back scattering in Varian EPID [16]. To remove the non-uniform backscattering pattern in calibration process, the impact of the backscatter that was present during the flood-field calibration was deduced and, then, the estimated non-uniform back-scatter pattern is corrected [17]. The absolute dose of the transit beam was first measured with EPID in calibrated units (CU) and converted to the absorbed dose. Figure 2(a) shows the experimental setup for EPID calibration with ion chamber measurement where we used 10 × 10 cm^2^ field size and 20 cm-thick homogeneous phantom as a simple approximation of field size and patient thickness, respectively. Both Eclipse calculation and measurement were done in 8 mm depth [8]. To convert the CU to dose, the calibration curve was acquired by comparison between dose measured by ion chamber and CU measured by EPID signal. Figure 2(b) shows that CU vs. dose on the beam axis as a function of CU (i.e., beam-on time). Although it seems like there is a linear relationship between dose and CU, the relationship is non-linear at low CU values. Figure 2(c) shows that the absorbed dose per CU decreases as the CU values increases and then it becomes relatively constant. Using this conversion factor, the measured EPID signal was converted to the absorbed dose. The data produced by the TPS and EPID were compared using the RIT 113 software module (Radiological Imaging Technology, Ver. 5.2, Colorado Springs, CO, USA) which usually takes less than 3 seconds for each field.

The gamma evaluation method was used to compare dose distributions with 3% dose difference and 3 mm distance-to-agreement (DTA) criteria [18]. The gamma index (GI) value of a point on the image can be calculated using the equation:

1 $$ {\Gamma}_{\overrightarrow{r_r}}\left(\overrightarrow{r_e}\right)\kern0.5em =\sqrt{\frac{{r_{\overrightarrow{r_r}}}^2\left(\overrightarrow{r_e}\right)}{\varDelta {d}^2}+\frac{{\delta_{\overrightarrow{r_r}}}^2\left(\overrightarrow{r_e}\right)}{\varDelta {D}^2}} $$

2 $$ \gamma \left({\overrightarrow{r}}_r\right)= \min \left\{\varGamma \left({\overrightarrow{r}}_r,{\overrightarrow{r}}_e\right)\right\}\forall \left\{{\overrightarrow{r}}_e\right\} $$

where, $\overrightarrow{r_e}$ and $\overrightarrow{r_r}$ are the position vectors of the pixel in the reference image and the evaluation image, respectively; ${r}_{\overrightarrow{r_r}}$ represents the distance between the positions; and ${\delta}_{\overrightarrow{r_r}}$ denotes the difference in dose at each position [18].

## Results

**Figure 3:**
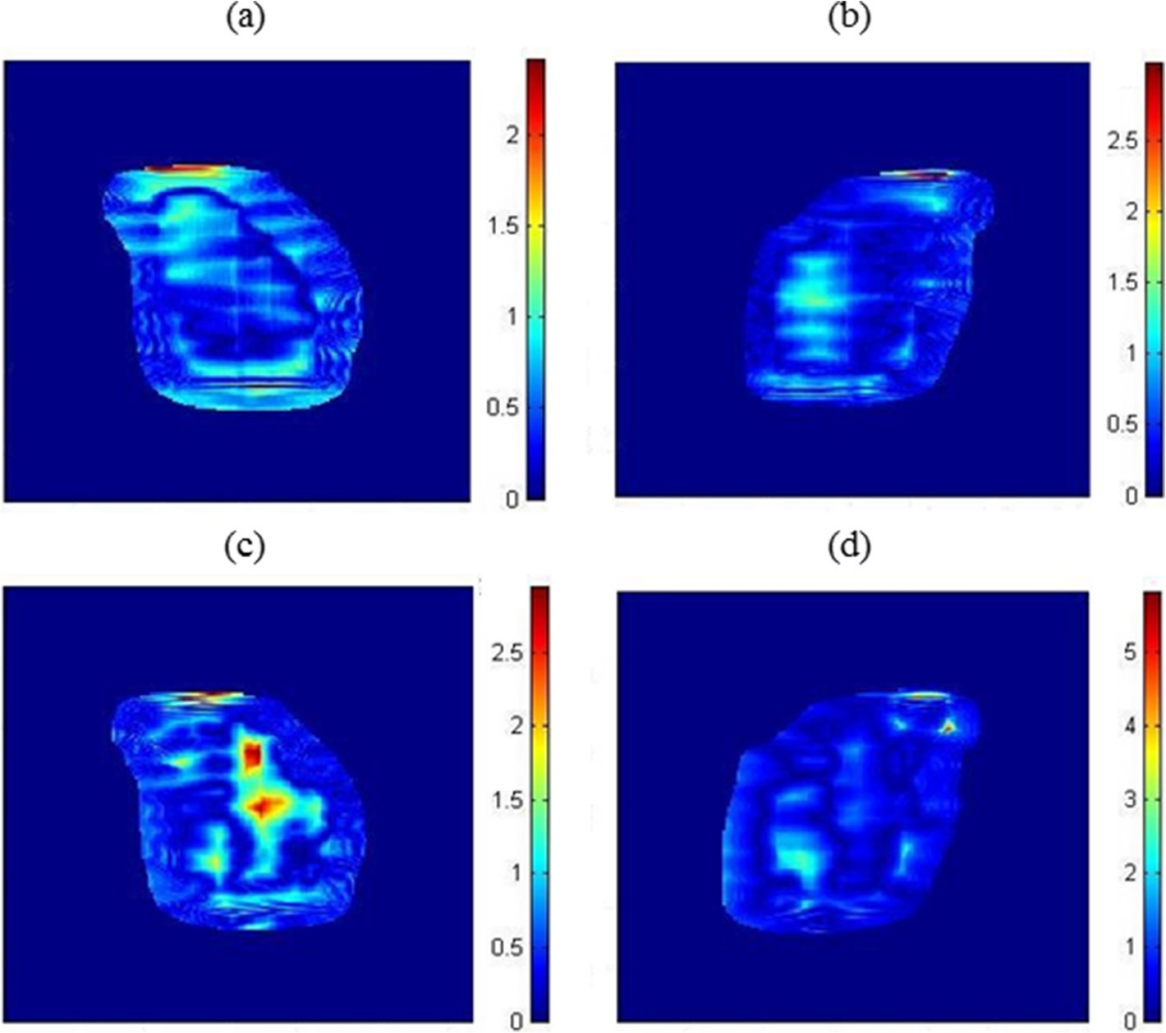
**Transit dose comparisons for fields 1 and 5 of patient 2. (a)** field 1 with a homogeneous phantom, **(b)** field 5 with a homogeneous phantom, **(c)** field 1 with an anthropomorphic phantom and **(d)** field 5 with an anthropomorphic phantom.

The effectiveness of the proposed method was first evaluated using a 20 cm-thick homogeneous plastic phantom. Figure 3(a) and (b) show the GI maps of transit doses passing through a homogeneous solid water phantom for fields 1 and 5 of patient 2 in Table 1, respectively. The passing rates were 96.6% and 95.4%, respectively, indicating that the transit dose map measured with the EPID is well matched with the dose distribution calculated by the TPS. This finding suggested that the gamma index-based comparison of measured and TPS calculated transit dose can be used to prevent accidents in radiotherapy since the passing rate will decrease significantly if errors occur during treatment. That is, the proposed method can effectively assess the errors of the treatment plan at the final stage (i.e., in the treatment room). Table 2 shows the detailed passing rate for the 24 IMRT fields, revealing an average normal beam delivery of 94.2%.

**Table 2:** **Gamma index (3%/3 mm)-based passing rates for transit dose measured with an homogeneous phantom**

	**Patient 1 (%)**	**Patient 2 (%)**	**Patient 3 (%)**	**Patient 4 (%)**
Field 1	92.6 (8.6 × 7 cm^2^)	96.6 (6.8 × 4.5 cm^2^)	92.0 (8.6 × 6.1 cm^2^)	95.6 (6.6 × 5 cm^2^)
Field 2	92.7 (8.3 × 7 cm^2^)	95.5 (6.3 × 4.8 cm^2^)	95.0 (7.8 × 6.3 cm^2^)	95.1 (6.3 × 5 cm^2^)
Field 3	90.8 (8.7 × 7 cm^2^)	95.7 (6.6 × 4.8 cm^2^)	96.5 (6.6 × 6.3 cm^2^)	95.6 (5.6 × 5 cm^2^)
Field 4	91.3 (9.3 × 7 cm^2^)	95.3 (6.6 × 4.8 cm^2^)	93.6 (6.5 × 6.3 cm^2^)	95.3 (5.9 × 5 cm^2^)
Field 5	92.7 (8.6 × 7 cm^2^)	95.4 (7.1 × 4.8 cm^2^)	93.1 (8.3 × 6.1 cm^2^)	95.4 (6.6 × 5 cm^2^)
Field 6	91.9 (8.6 × 7 cm^2^)	97.4 (6.3 × 4.8 cm^2^)	90.1 (8.3 × 6.1 cm^2^)	95.8 (5.8 × 5 cm^2^)
Mean	92.0	96.0	93.4	95.4
SD	0.8	0.9	2.2	0.3

Parenthesis under the passing rates denotes the collimator (jaw) size of each beam.

GI analysis of the calculated transit doses for fields 1 and 5 of patient 2 using the anthropomorphic phantom, however, showed more regions with GI greater than 1, indicating an increased percentage of failure (Figure 3(c), (d)). The passing rates were decreased to 84.9% and 94.4%, respectively. Detailed gamma analysis of transit dosimetry with anthropomorphic phantoms showed that the passing rates are generally decreased, to a mean ± SD of 86.8% ±3.8% (Table 3). Table 3 also shows the absolute dose difference (ADD) at the beam center between the TPS calculated and EPID measured values. The mean ± SD percentage difference was −0.38% ± 2.1%, showing that the absorbed doses of the transit beam measured with the EPID were close to the doses calculated by the TPS.

**Table 3:** **Comparison of absolute and relative transit doses measured with EPID and the lung region of an anthropomorphic phantom**

**Field**	**Patient 1**		**Patient 2**		**Patient 3**		**Patient 4**	
	**ADD (%)**	**RDP (%)**	**ADD (%)**	**RDP (%)**	**ADD (%)**	**RDP (%)**	**ADD (%)**	**RDP (%)**
Field 1	−1.1	83.3	−1.4	84.9	0.1	87.0	−1.4	86.6
Field 2	−0.8	85.7	2.2	90.3	3.9	87.2	−0.6	86.7
Field 3	−4.0	89.5	−1.4	87.9	0.5	88.8	1.1	88.9
Field 4	0.6	82.2	−0.7	82.4	−1.2	87.3	−5.1	92.2
Field 5	−1.3	83.6	1.0	94.4	−3.3	91.2	3.5	93.8
Field 6	−2.2	79.3	0.2	81.8	0.8	80.4	1.7	87.8
Mean	−1.5	83.9	0.0	86.9	0.1	87.0	−0.1	89.3
SD	1.5	3..4	1.4	4.9	2.4	3.6	3.0	3.0

*Abbreviations*: *ADD* absolute dose difference, defined as percentage dose difference at the beam center, *RDP* relative dose passing rate (RDP), defined as gamma index (3%/3 mm)-based passing rate.

It is reasonable that the reduced passing rate with anthropomorphic phantoms comes from the inaccuracy of TPS calculations for inhomogeneity and the limitations of the analysis of the measurement results, ignoring a number of correction factors. To prove whether inaccuracy of the heterogeneity correction algorithm in Eclipse is more dominant in causing the observed deviations between measured and predicted dose distributions for lung treatments, additional experiments in the pelvic region of the anthropomorphic phantom was carried out. Table 4 shows the passing rates about the IMRT fields from patient 1 and 2. As seen in Table 4, the passing rates are much higher than the values in Table 3 but lower than the values in Table 2. This result suggests that, although the calculation algorithm of the TPS worked well for the homogeneous phantom, its accuracy was reduced for inhomogeneous material.

**Table 4:** **Comparison of relative transit doses measured with EPID and the pelvic region of an anthropomorphic phantom**

	**Field 1**	**Field 2**	**Field 3**	**Field 4**	**Field 5**	**Field 6**	**Mean**
Passing rate (%) of patient 1	96.6	90.7	90.3	93.1	89.8	90.5	91.8
Passing rate (%) of patient 2	93.9	95.1	93.6	94.6	94.0	92.6	94.0

## Discussion

We have measured the transit doses of 24 IMRT fields with EPID and compared them with the doses calculated from the TPS. Comparisons of absolute doses with the anthropomorphic phantom showed good agreement between measured and calculated doses at the beam center. Although the relative doses of the measured and calculated transit beams passing through a *homogeneous* phantom were well matched, the average passing rate was reduced to 86.8% when using an *inhomogeneous* anthropomorphic phantom.

In general, the EPID response is a function of various factors such as field size, phantom thickness, lateral scatter in the EPID, phantom-to-EPID scatter and the measured dose will generally have relatively large uncertainties if one omits corrections for all these effects. The dependence of EPID response on various factors and corresponding calibration method were studied previously and discussed extensively by Nijsten et al. [19]. Although many research groups have reported and suggested the calibration methods of EPID for various factors, it is still too complex to apply this method clinically and need more simple way to do it. Our experimental results suggest that, based on the TPS and EPID measurement with simple calibration, we can monitor (or verify) whether the desired beam (or dose fluence) was delivered to the patient without large error during treatment. If one is interested in detection for this kind of large error, rough calibration of EPID seems to work well.

**Figure 4:**
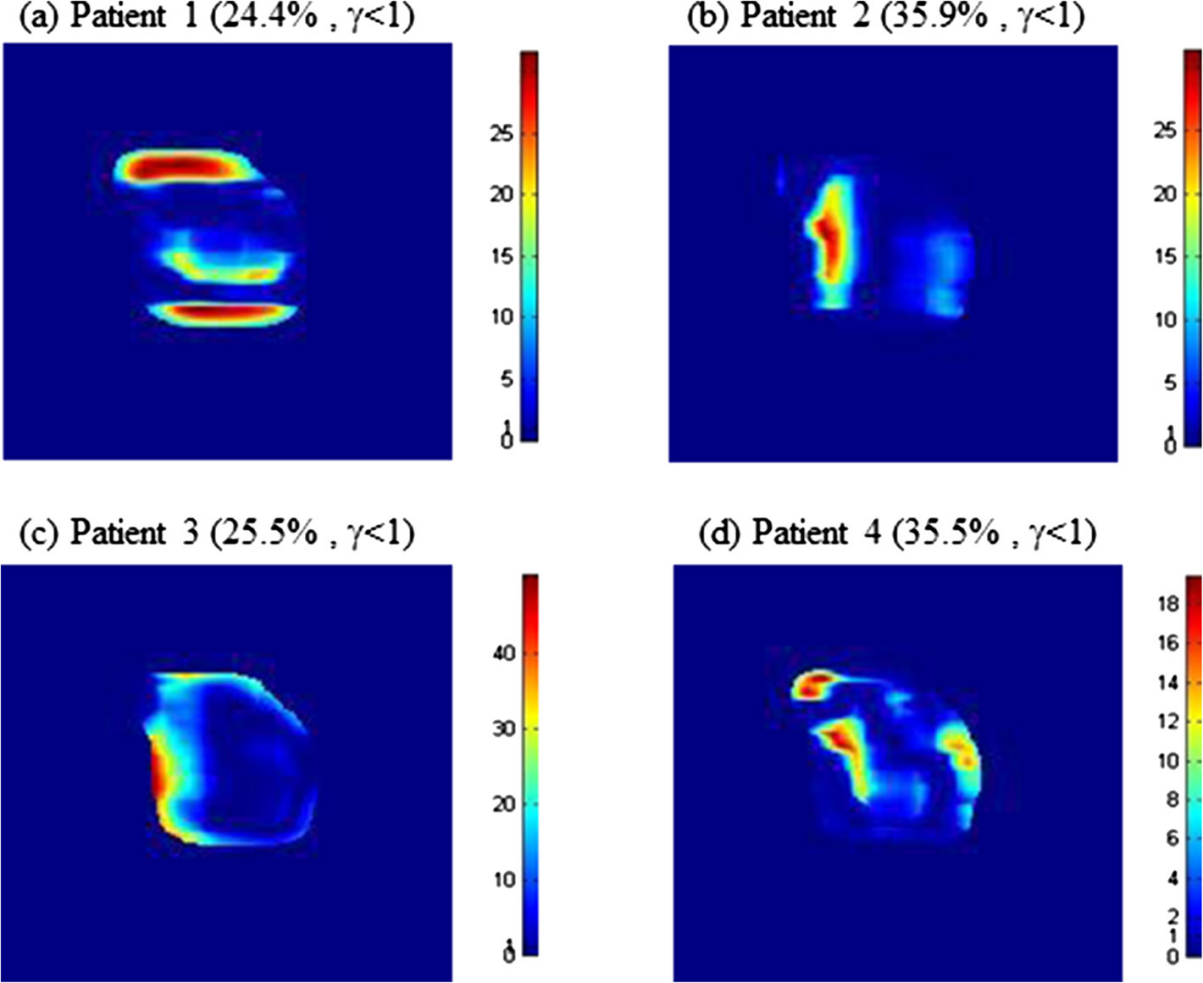
**Simulation results for change in gamma index when erroneously choosing wrong fields rather than field 1 of patient 2. (a)** Field 2 of patient 1, **(b)** field 2 of patient 2, **(c)** field 2 of patient 3 and **(d)** field 2 of patient 4.

To evaluate whether the proposed transit dosimetry is feasible in detecting large errors during treatment, we deliberately delivered radiation to the wrong IMRT fields and evaluated changes in transit dose distribution (Figure 4). For example, when field 2 of patient 2 was erroneously irradiated with the dose planned for field 1 of patient 2, the passing rate of the gamma index was decreased to 35.9% (Figure 4(b)). This result would thus show an error in beam delivery since the passing rate would be too low compared with normal beam delivery. Table 5 shows the passing rates when either the wrong patient or the wrong field was chosen erroneously. Choosing the wrong fields significantly decreased the GI passing rate for the transit dose, to an average of 30.0%. These findings indicate that transit dosimetry can be used to detect beam delivery errors since changes in transit dose passing through a patient can be easily detected.

**Table 5:** **Gamma index (3%/3 mm)-based passing rates for transit dose measured with EPID and an anthropomorphic phantom following selection of the wrong fields instead of field 1 of patient 2**

**Fields**	**Patient number**			
	**Patient 1 (%)**	**Patient 2 (%)**	**Patient 3 (%)**	**Patient 4 (%)**
Field 1	20.5	-	32.4	38.4
Field 2	24.4	35.9	25.5	35.5
Field 3	31.0	49.7	30.1	49.3
Field 4	18.9	32.9	16.9	29.0
Field 5	35.0	37.8	14.9	21.6
Field 6	22.1	32.2	15.6	31.5
Mean	**25.3**	**37.7**	**22.6**	**34.2**

**Figure 5:**
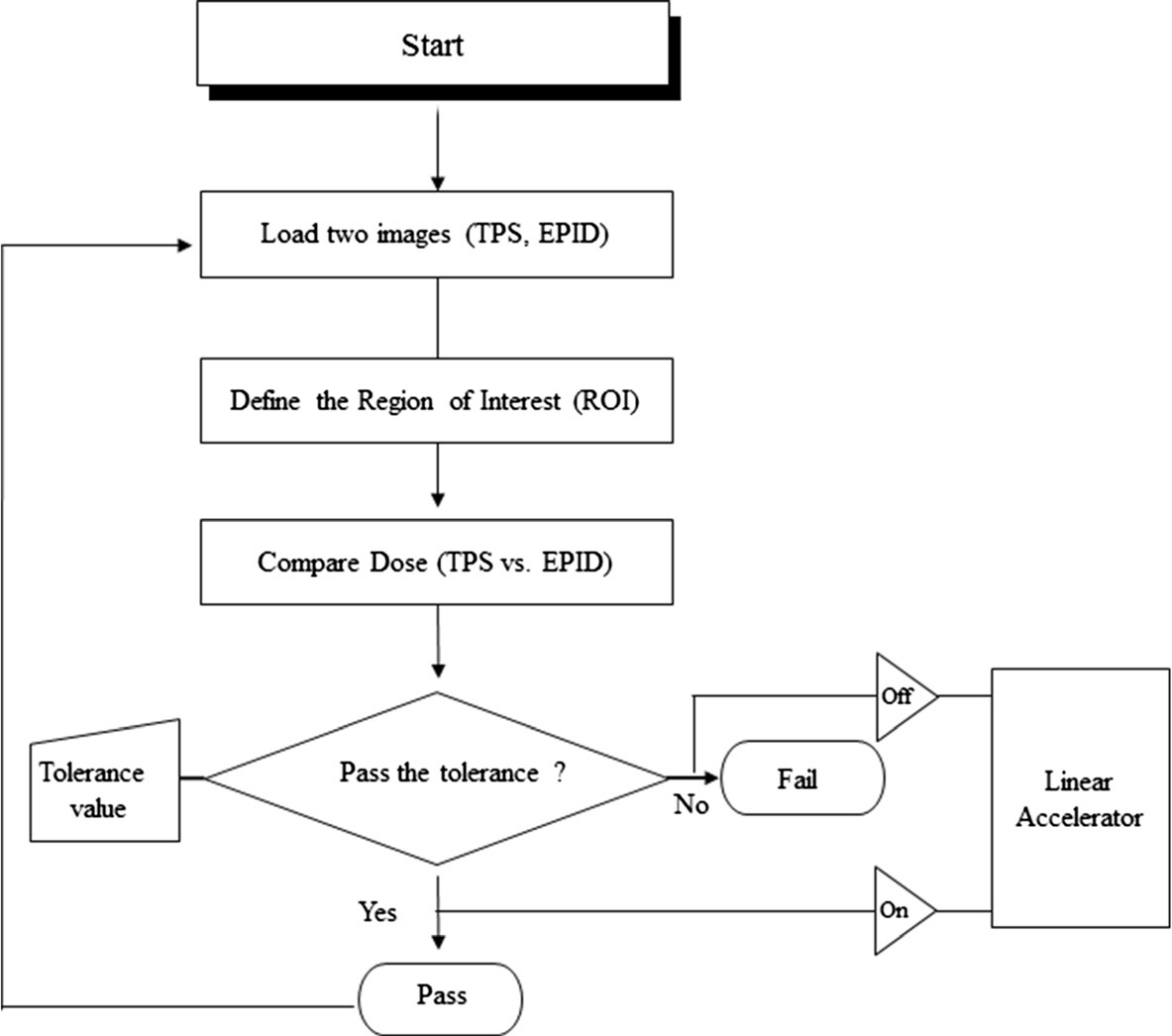
**Flow chart for the verification of radiotherapy beam in the treatment room using comparisons of transit doses.**

Figure 5 shows an example of flow chart for the treatment beam monitoring using comparisons of the transit dose. The first step is to obtain calculated and measured dose maps from TPS and EPID, respectively. This is followed by defining a common rectangular region of interest (ROI) for later comparison of dose correlations. In the next step, doses are compared based on gamma index (GI) analysis. If these comparisons pass the predefined tolerance value, the treatment proceeds to the next field; if not, the treatment should be stopped and the reason for the mismatch should be verified. Although the proposed method is simple, it needs further refinement as a future study since the EPID signal should be converted to absorbed dose using actual treatment field size and patient thickness.

## Conclusion

We have compared EPID measurements of transit dose for 24 IMRT fields with doses calculated from TPS. Our feasibility study suggests that EPID based transit dose monitoring in the treatment room can provide information about the accuracy of beam delivery.

## References

[1] Langer M, Brown R, Urie M, Leong J, Stracher M, Shapiro J (1990). Large scale optimization of beam weights under dose-volume restrictions. Int J Radiat Oncol Biol Phys.

[2] Langer M, Leong J (1987). Optimization of beam weights under dose-volume restrictions. Int J Radiat Oncol Biol Phys.

[3] Morrill SM, Lane RG, Wong JA, Rosen II (1991). Dose‐volume considerations with linear programming optimization. Med Phys.

[4] *ORH information notice 2005-01*; 005. [http://www.health.ny.gov/environmental/radiological/radon/radioactive_material_licensing/docs/berp2005_1.pdf]

[5] Williams M (2007). Radiotherapy near misses, incidents and errors: radiotherapy incident at Glasgow. Clin Oncol.

[6] Commission USNR: **Gamma knife treatment to wrong side of brain.** *Event Notification Report* 2007, **43746**.

[7] Ash D (2007). Lessons from epinal. Clin Oncol.

[8] Van Esch A, Depuydt T, Huyskens DP (2004). The use of an aSi-based EPID for routine absolute dosimetric pre-treatment verification of dynamic IMRT fields. Radiother Oncol.

[9] Van Esch A, Vanstraelen B, Verstraete J, Kutcher G, Huyskens D (2001). Pre-treatment dosimetric verification by means of a liquid-filled electronic portal imaging device during dynamic delivery of intensity modulated treatment fields. Radiother Oncol.

[10] Essers M, Boellaard R, van Herk M, Lanson H, Mijnheer B (1996). Transmission dosimetry with a liquid-filled electronic portal imaging device. Int J Radiat Oncol Biol Phys.

[11] Reich P, Bezak E, Mohammadi M, Fog L (2006). The prediction of transmitted dose distributions using a 3D treatment planning system. Australas Phys Eng Sci Med.

[12] Mohammadi M, Bezak E (2006). Two-dimensional transmitted dose measurements using a scanning liquid ionization chamber EPID. Phys Med Biol.

[13] McNutt TR, Mackie TR, Reckwerdt P, Papanikolaou N, Paliwal BR (1996). Calculation of portal dose using the convolution/superposition method. Med Phys.

[14] Mohammadi M, Bezak E, Reich P (2006). Comparison of two-dimensional transmitted dose maps: evaluation of existing algorithms. Australas Phys Eng Sci Med.

[15] van Elmpt W, McDermott L, Nijsten S, Wendling M, Lambin P, Mijnheer B (2008). A literature review of electronic portal imaging for radiotherapy dosimetry. Radiother Oncol.

[16] Rowshanfarzad P, McCurdy BM, Sabet M, Lee C, O’Connor DJ, Greer PB (2010). Measurement and modeling of the effect of support arm backscatter on dosimetry with a Varian EPID. Med Phys.

[17] Vinall A, Williams A, Currie V, Van Esch A, Huyskens D (2010). Practical guidelines for routine intensity-modulated radiotherapy verification: pre-treatment verification with portal dosimetry and treatment verification with in vivo dosimetry. Br J Radiol.

[18] Low DA, Harms WB, Mutic S, Purdy JA (1998). A technique for the quantitative evaluation of dose distributions. Med Phys.

[19] Nijsten S, Van Elmpt W, Jacobs M, Mijnheer B, Dekker A, Lambin P, Minken A (2007). A global calibration model for a‐Si EPIDs used for transit dosimetry. Med Phys.

